# Global trends of Pauwels type III femoral neck fractures: bibliometric analysis and visualized study

**DOI:** 10.3389/fsurg.2024.1372310

**Published:** 2024-09-16

**Authors:** Mengyu Wang, Ze Zhang, Fengpo Sun, Yi Zhu, Ruining Han, Zijie Pei, Zhaoshuo He, Junzhi Liu, Liangyuan Wen

**Affiliations:** ^1^ Department of Orthopedics, Beijing Hospital, National Center of Gerontology, Institute of Geriatric Medicine, Chinese Academy of Medical Sciences, Beijing, China; ^2^ The Fifth Clinical Medical College of Perking University, Beijing, China; ^3^ Beijing Hospital, National Center of Gerontology, Institute of Geriatric Medicine, Chinese Academy of Medical Sciences &Peking Union Medical College, Beijing, China

**Keywords:** femoral neck fracture, pauwels type III, internal fixation, visualized study, bibliometrics, global trends

## Abstract

**Background:**

Pauwels type III femoral neck fractures, as a serious type of femoral neck fractures, have brought about a heavy economic burden on families and society for the high disability rate. Through bibliometric research and visualized analysis, this study aimed at elucidating the global research status of Pauwels type III femoral neck fractures to date, and predicting the future research trends in this field.

**Methods:**

Publications and associated information on Pauwels type III femoral neck fractures to date were retrieved from Web of Science Core Collection, and by VoSviewer and R package “bibliometrix”, bibliometric analysis and visual presentation was conducted.

**Results:**

By retrieval, a total of 98 studies were refinedly extracted, and the volume of publications in this field increased year-over-year. China ranked first in terms of total publication volume and H-Index, with its total citation records second only to the United States. The country with the highest average citation frequency was Switzerland. SHANGHAI JIAO TONG UNIVERSITY was the most productive research institution. Among the authors in this field, Li, Jiantao had published the most researches. *INJURY INTERNATIONAL JOURNAL OF THE CARE OF THE INJURED* and *JOURNAL OF ORTHOPAEDIC TRAUMA* were the two magazines with the highest publication volume, total citation records, and H-index. According to keywords co-occurrence analysis, the research content in the past 24 years is mainly divided into four different dimensions. Finite element analysis, femoral neck system, medial buttress plate, cannulated screws, hip screw, open reduction, complications are hot topics for future research.

**Conclusions:**

According to the global trends analysis of publications production, Pauwels type III femoral neck fractures are receiving increasing attention and input from scholars. China has made the greatest scientific research contribution among countries, but its academic quality should be improved further. The modified therapeutic methods designed for addressing the complications of traditional internal fixation for Pauwels III femoral neck fractures will be the future research hotspot.

## 1 Introduction

Femoral neck fracture is one of the most common fractures, accounting for 50% of hip fractures (1). Based on the angle between the fracture line and the straight line perpendicular to the long axis of the femur, known as the Pauwels angle (2–4), femoral neck fractures were divided into Pauwels type I (Pauwels angle ≤30°), Pauwels type II (Pauwels angle >30° and ≤50°), and Pauwels type III (Pauwels angle >50°). Pauwels type III femoral neck fractures are usually caused by high-energy injuries, and are more common in young people. A meta-analysis has indicated that the nonunion rate of Pauwels III femoral neck fractures is as high as 33% as well as the avascular necrosis rate is up to 16% (5), due to high-energy violence, poor biological environment and severe damage of femoral head blood supply. As a result of the high disability rate, Pauwels type III femoral neck fractures have brought about a heavy economic burden on families and society.

The cornerstone of treatment for Pauwels type III femoral neck fractures in young people is anatomical reduction and stable internal fixation (6), of which the most commonly used internal fixation methods in clinical practice are multiple cannulated screws fixation and dynamic hip screw fixation. Although angle stabilization devices represented by dynamic hip screw have biomechanical advantages over cannulated screws, they typically have higher invasiveness and more soft tissue detachment, leading to an increased rate of reoperation. Therefore, cannulated screws are still the most common treatment method for femoral neck fractures in young people, but the incidence of postoperative internal fixation failure, fractured fragments displacement, femoral neck shortening, varus deformity, and nonunion is relatively high (7, 8). In recent years, the internal fixation therapeutic methods for Pauwels type III femoral neck fractures have been continuously explored, but there's still no consensus over the optimal treatment plan in academic circles.

Bibliometrics is a literature analysis method that analyzes the output and status of publications in specific research fields from both quantitative and qualitative perspectives (9, 10), effectively demonstrating the development of the research field. At present, the global trends analysis of Pauwels type III femoral neck fractures is not yet clear. Therefore, from Web of Science Core Collection (WoSCC) database, we used bibliometric analysis methods to obtain information related to publications in this field, and visualized the detailed information through bibliometric tools such as VoSviewer and R package “bibliometrix”. This study aimed at elucidating the global research status of Pauwels type III femoral neck fractures to date, and predicting the future research trends in this field, so as to provide some suggestions for treatment decisions and related medical policies for Pauwels type III femoral neck fractures.

## 2 Methods

### 2.1 Data sources and search strategy

**Figure 1:**
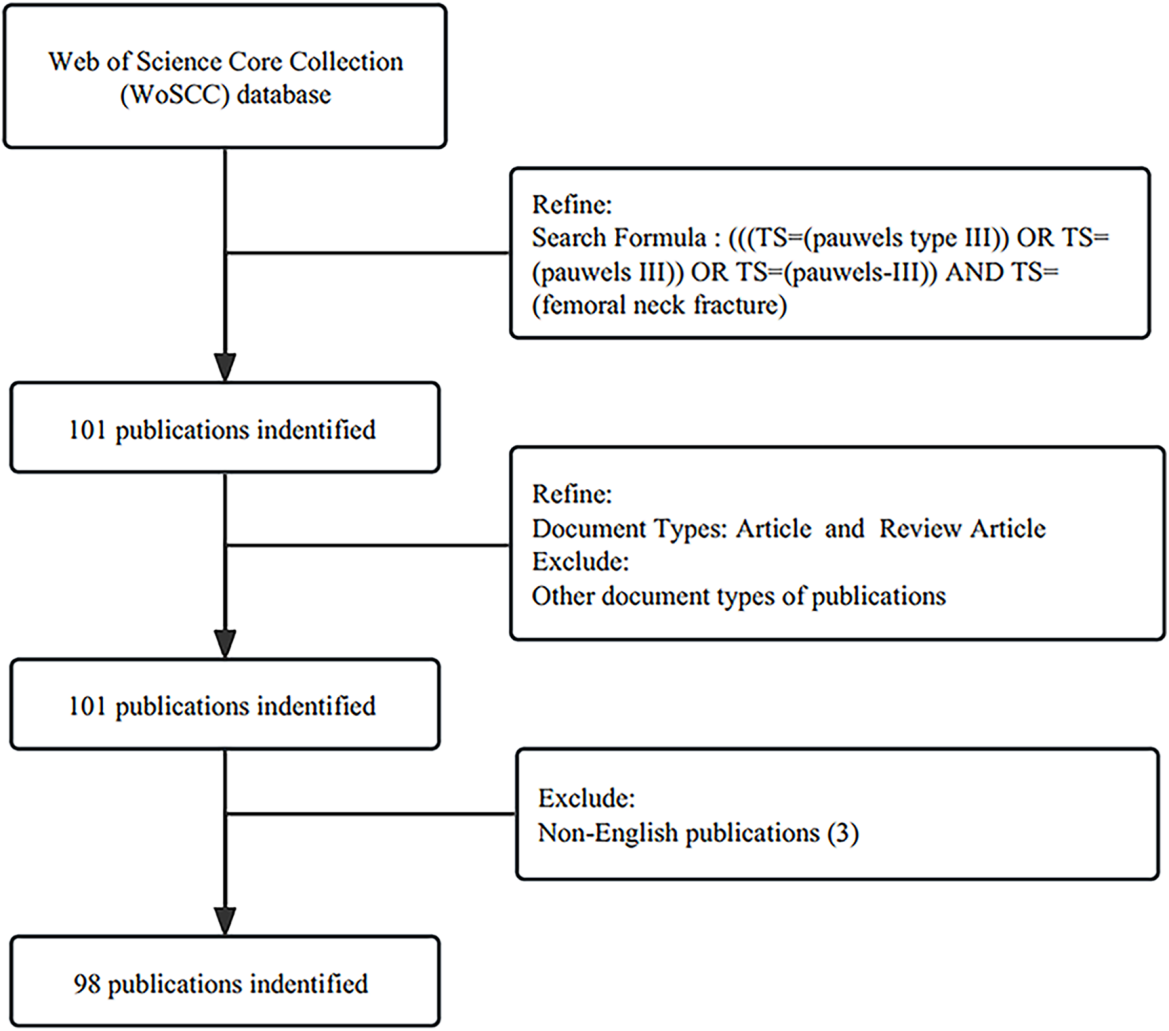
Literature retrieval and extraction strategy of this study. This study searched for all publications that meet the requirements to date, spanning from 1999 to 2023.

The literature search was conducted in the Web of Science Core Collection (11) (WoSCC) database on July 7, 2023. The WoSCC database is a valuable source for bibliometric analysis, featuring prominent academic journals and international conference records from diverse research fields. The search formula was “(([TS = (pauwels type III)] OR TS = (pauwels III)) OR TS = (pauwels-III)) AND TS = (femoral neck fracture)”, and we restricted document types to articles and review articles in English. The time span of all publications that met the search requirements to date was from 1999 to 2023. Full records and references of the identified publications were exported to a plain text file (Figure 1).

In this study, the publication year, number of publications, author, journal, country, institution, total citations, average citations per term, impact factor, and H-index were exclusively retrieved from the WoSCC database. The H-index, proposed by Hirsch in 2005, is a comprehensive and quantitative metric at the author level that indicates the productivity and citation impact of an individual author's publications. To ensure the accuracy of the metadata, the retrieval and screening process was independently conducted by 2 authors and reviewed by a third author for quality control. A total of 98 publications were meticulously extracted and exported in plain text format for analysis.

### 2.2 Statistical analysis

VOSviewer (version 1.6.17, Leiden University), is an advanced knowledge domain mapping software used for constructing and visualizing bibliometric networks, extracting key information from complex publications, and conducting bibliometric analysis, as well as establishing co-authorship, co-citations, bibliographic coupling, and co-occurrence networks.

In the networks of VOSviewer, node size represents the occurrence of items found using various analysis methods, and items are color-coded into different clusters. Weighted total link strength (TLS) lines were used to visualize the associations between authors, institutions, and countries, which is based on the total co-occurrence of keywords. The link's thickness in the visual analysis increases with higher TLS values.

In R (Version 4.3.2), the “bibliometrix” package is an open-source tool for quantitative research in scientometrics and bibliometrics, encompassing all the primary bibliometric methods of analysis. The Bibliometrix package could conduct bibliometric analysis, and construct data matrices for co-citation, coupling, scientific collaboration analysis, and co-word analysis. Microsoft Office Excel 2022 was used to quantitatively analyze the publications by collecting and ranking all the publication characteristics, as well as calculating the respective proportions based on the total number of 98 publications. Additionally, the predicted publication growth model equation was derived as a binomial function using the built-in capabilities of Microsoft Office Excel 2022.

## 3 Results

### 3.1 Number of publications each year

**Figure 2:**
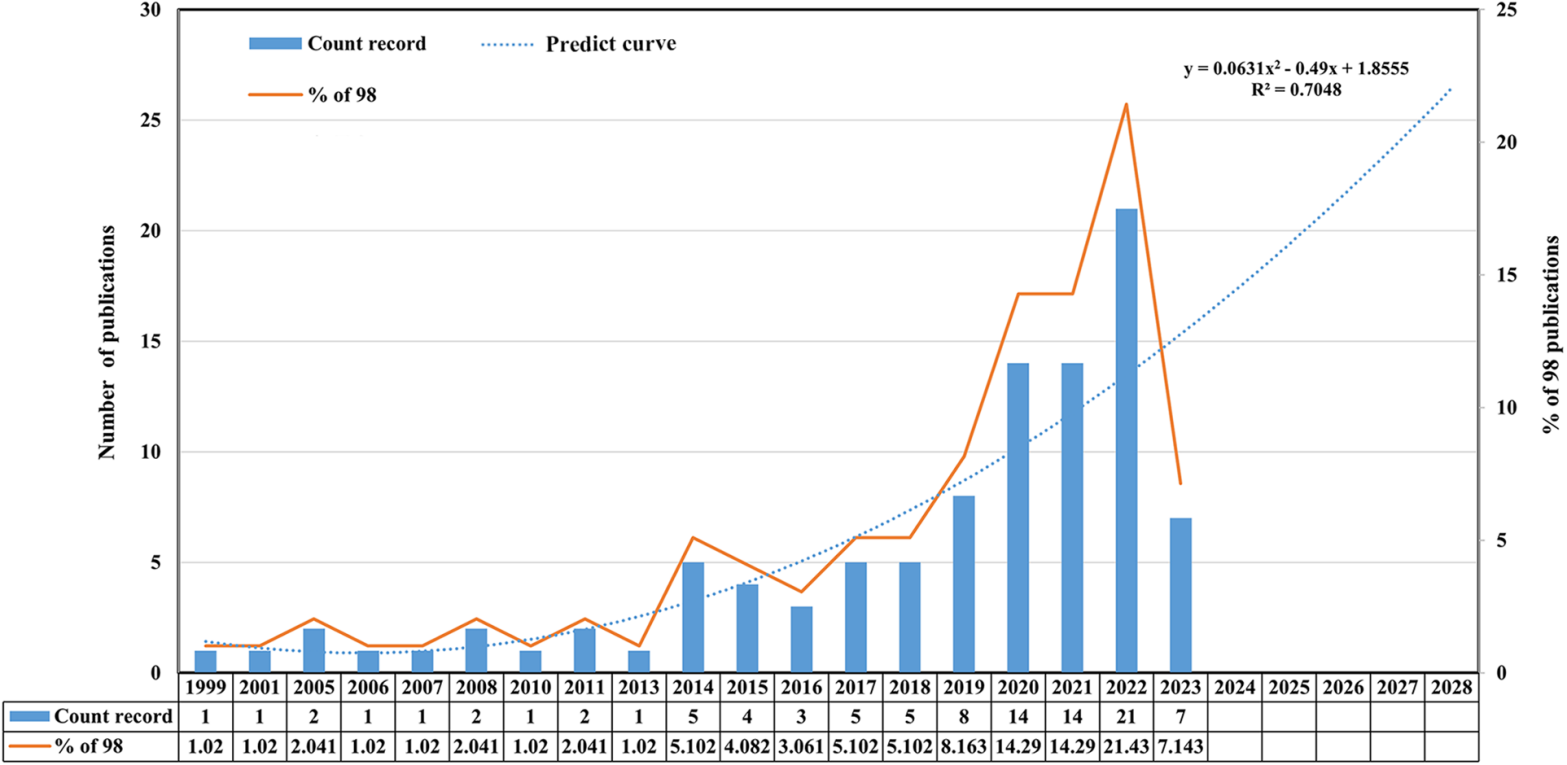
Annual publication volume and proportion in this field from 1999 to 2023. This study analyzed the publication volume information as of July 7, 2023. So the number of publications in 2023 shown in the figure was relatively low and incomplete.

The number of publications concerning Pauwels III femoral neck fractures was gradually increasing. In Figure 2, the publication volume in 2022 reached its peak (*n* = 21, 21.73%) among all complete years of production in the past. Besides, according to the predicted growth model equation, *y *= 0.0631 *× *^2^–0.49*x + *1.855, *R*^2^ = 0.7048, in which *x* represented the year, and y stood for the predicted number of publications each year, the publication number of 2028 was estimated to be more than 25.

### 3.2 Production and collaboration of each country

In order to make the publications number of each country visualized, we sorted out and ranked the product distribution of each country. In Table 1, as to the publication volume and proportion by country, CHINA came the first (*n* = 45, 45.92%), USA ranked the second (*n* = 16, 16.33%), and the third was GERMANY (*n* = 9, 9.18%). And the top 3 countries for H-Index were also mentioned above. Besides, as to the total citation records, the top 3 countries were USA (*n* = 480), CHINA (*n* = 386), and GERMANY (*n* = 245), far surpassing other countries. However, regarding to the average citation, the top 3 countries were SWITZERLAND (*n* = 42.7), USA (*n* = 30.0) and GERMANY (*n* = 27.2). Unlike before, CHINA got 8.6 average citations, ranking eighth.

**Table 1:** The top 10 countries with the highest publication volume about Pauwels III femoral neck fractures.

Rank	Country/region	Number of publications	Proportion (%)	Citation record			H-Index
				Total	Without self-citations	Average citation	
1	China	45	45.92	386	317	8.6	11
2	USA	16	16.33	480	454	30.0	8
3	Germany	9	9.18	245	241	27.2	6
4	Brazil	6	6.12	33	33	5.5	3
5	India	5	5.10	45	44	9.0	3
6	South Korea	4	4.08	40	37	10.0	3
7	Austria	4	4.08	46	46	11.5	3
8	Taiwan	4	4.08	65	64	16.3	4
9	Switzerland	3	3.06	128	127	42.7	2
10	Turkey	3	3.06	1	1	0.3	1

*This ranking is based on the number of publications by country or region.

**Figure 3:**
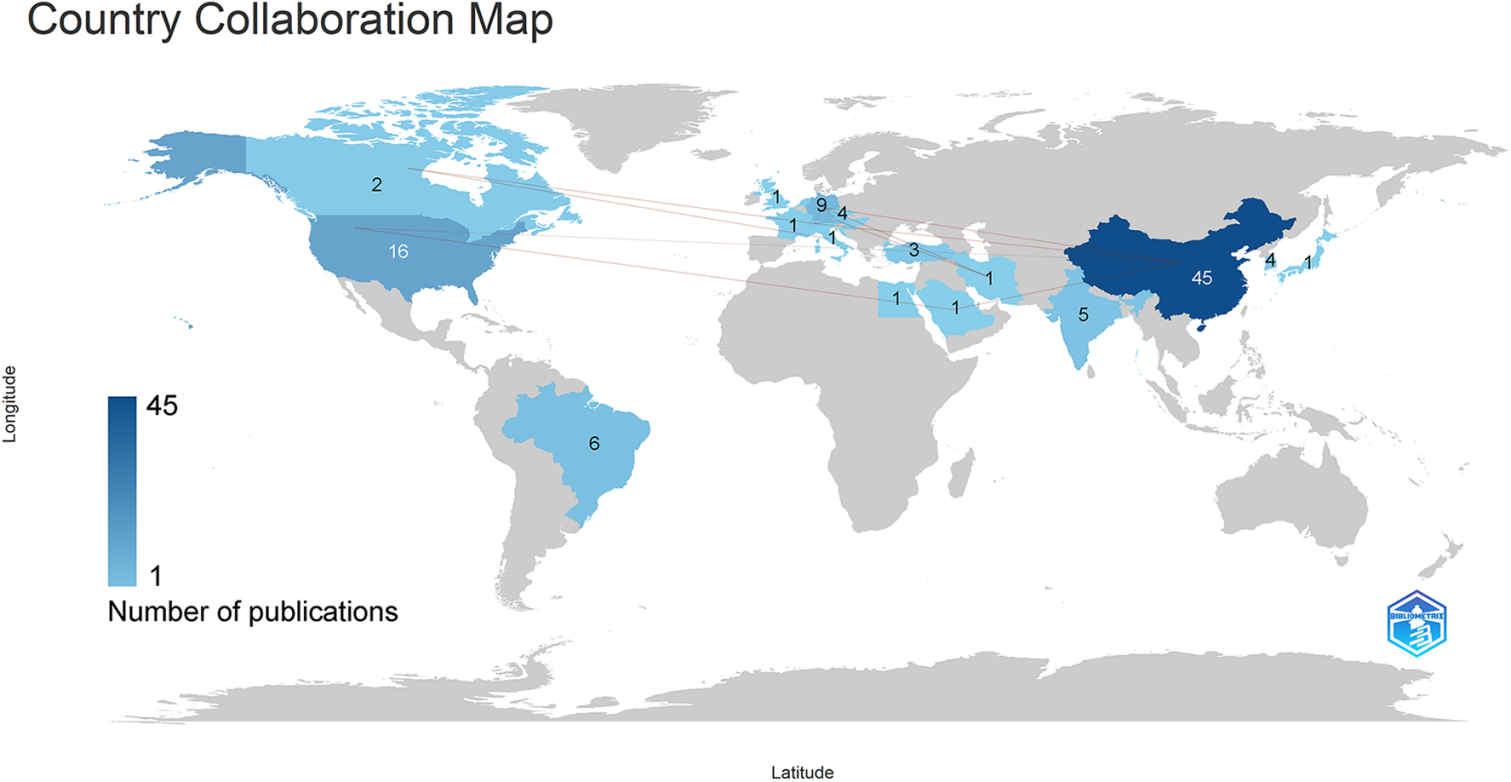
Global distribution of publication volume and country collaboration about Pauwels III femoral neck fractures research. By 2023, all countries or regions that had published relevant research in this field had been marked with varying shades of blue, while countries marked in gray had not yet published such research.

Following Figure 3, simply, there were 22 countries or regions had published publications about Pauwels III femoral neck fractures. What's more, the depth of colors on each country in the map reflected their respective publication volume and the darker the color was, the more the country had published. The lines in the map briefly stood for the collaboration about relevant publications among these countries. From Figure 3, it was visualized that CHINA was the most productive country in this field, with the darkest color. Moreover, CHINA had established cooperation with the vast majority of countries in this field such as USA, GERMANY, CANADA and so on.

### 3.3 The most productive affiliations and funding agencies involved in

By 2023, more than 200 affiliations had published the relevant research, and the most productive 10 affiliations all over the world were showed in Table 2. Shanghai Jiao Tong University, affiliated to CHINA, took the lead with 6 publications. Moreover, there were more than half of the top 10 affiliations belonging to CHINA.

**Table 2:** The most productive 10 affiliations publishing the related researches.

Rank	Affiliations	Records	Country
1	Shanghai Jiao Tong University	6	China
2	Chinese Peoples Liberation Army General Hospital	5	China
3	Peking University	4	China
4	Tianjin Hosp	4	China
5	Army Medical University	3	China
6	Eulji University	3	South Korea
7	Friedrich Schiller University of Jena	3	Germany
8	Home	3	Brazil
9	Kettering University	3	USA
10	Soochow University China	3	China

Table 3 simply demonstrated the top 5 most supportive funding agencies in this field. National Natural Science Foundation Of China, belonging to CHINA had 13 support records in the research area, accounting for 13.27%, outnumbering any other funding agency across the world. China Postdoctoral Science Foundation (*n* = 2, 2.04%), Interdisciplinary Program Of Shanghai Jiao Tong University (*n* = 2, 2.04%) and Mclaren Foundation Of Flint Michigan (*n* = 2, 2.04%) shared the second. Significantly, it was noteworthy that the top 3 funding agencies were all from CHINA, while the other 2 were both from USA.

**Table 3:** The top 5 most supportive funding institutions in the research field.

Rank	Funding agency	Records	Proportion (%)	Country
1	National Natural Science Foundation Of China	13	13.27	China
2	China PostdoctoralScience Foundation	2	2.04	China
3	Interdisciplinary ProgramOf Shanghai Jiao TongUniversity	2	2.04	China
4	Mclaren Foundation Of Flint Michigan	2	2.04	USA
5	Ao Foundation Via The Ao Technical Commission Trauma Network	1	1.02	USA

*This ranking is based on the records and proportion by funding agency.

### 3.4 The quality assessment of top journals and authors

By now, more than 40 journals reported the associated research in this field. And the most excellent 10 journals had published the most considerable publications, accounting for 59.41% of the whole. Moreover, some details about these journals could be visualized in Table 4. With the largest number of publications (*n* = 17), *Injury International Journal of the Care of the Injured* also got the highest H-index (H-index = 8). In addition, *Journal of Orthopaedic Trauma* got the most total citations (*n* = 368), which also ranked first about average citation. As to IF of 2022, *Computer Methods and Programs in Biomedicine* became the most influential one, with the impact factor as much as 6.1 in 2022.

**Table 4:** The top 10 excellent journals with the most considerable publications.

Rank	Journal	Number of publication	Total citation	Average citation	H-index	IF (2022)
1	Injury International Journal of the Care of the Injured	17	212	12.5	8	2.5
2	Journal of Orthopaedic Trauma	10	368	36.8	7	2.3
3	BMC Musculoskeletal Disorders	8	16	2.0	2	2.3
4	Archives of Orthopaedic and Trauma Surgery	6	75	12.5	3	2.3
5	Acta Ortopedica Brasileira	4	16	4.0	3	0.7
6	Journal of Orthopaedic Surgery and Research	4	72	18.0	3	2.6
7	Computer Methods and Programs in Biomedicine	3	41	13.7	3	6.1
8	Journal of International Medical Research	3	12	4.0	2	1.6
9	Orthopaedic Surgery	3	32	10.7	2	2.1
10	Bone & Joint Research	2	17	8.5	2	4.6

*This ranking is based on the number of publications by journal.

As was shown in Table 5, these top productive 10 authors around the world were Li, Jiantao(*n* = 5), Freitas, Anderson (*n* = 4), Li, Lianting(*n* = 3), Wang, M. -L. (*n* = 3), Maciel, Rafael Almeida. (*n* = 3), Martin S(*n* = 3), De Macedo Souto, Diogo Ranier(*n* = 3), Tang, Yong(*n* = 3), Wang, Gang(*n* = 3), Ma, Xinlong(*n* = 3). Li, Jiantao, who had achieved the most total citation records as well as H-Index was from CHINA, where more than half of the top 10 authors came from. What's more, Chinese author Li, Lianting and Wang, M. -L. had acquired the highest average citation (*n* = 19.33) in this field.

**Table 5:** The top 10 productive authors around the world in the field of research.

Rank	Author	Number of publications	Citation record			Country	H-Index
			Total	Without self-citations	Average citation		
1	Li, Jiantao	5	65	62	13.0	China	4
2	Freitas, Anderson	4	14	14	3.5	Brazil	2
3	Li, Lianting	3	58	58	19.3	China	3
4	Wang, M. -L.	3	58	58	19.3	China	3
5	Maciel, Rafael Almeida	3	13	13	4.3	Brazil	2
6	Martin S	3	27	25	9.0	USA	2
7	De Macedo Souto, Diogo Ranier	3	13	13	4.3	Brazil	2
8	Tang, Yong	3	14	12	4.7	China	2
9	Wang, Gang	3	14	13	4.7	China	2
10	Ma, Xinlong	3	19	19	6.3	China	1

*This ranking is based on the number of publications by author.

### 3.5 The top 10 landmark publications about Pauwels type III femoral neck fracture

In the 98 publications we retrieved from WoSCC database, we screened out the top 10 documents (2–4, 6, 12–17) with total citations at least 34. In Table 6, the document, written by Liporace, Frank in 2008, whose title was “Results of internal fixation of Pauwels type-3 vertical femoral neck fractures” earned the highest total citation (*n* = 187). “Pauwels’ classification of femoral neck fractures: Correct interpretation of the original”, written by Stoffel, Karl in 2017 ranked second subsequently, with the total citation (*n* = 120). And the third one was “Vertical shear fractures of the femoral neck—A biomechanical study” (*n* = 93), whose author was Baitner, Avi, written in 1999.

**Table 6:** The top 10 landmark publications about Pauwels type III femoral neck fractures.

Rank	Title	Investigator	Journal	Total citations (*n*)
1	Results of internal fixation of Pauwels type-3 vertical femoral neck fractures	Liporace, Frank et al. (14)	Journal of Bone and Joint Surgery-American Volume	187
2	Pauwels’ classification of femoral neck fractures: Correct interpretation of the original	Jan Bartonicek et al. (2)	Journal of Orthopaedic Trauma	120
3	Vertical shear fractures of the femoral neck—A biomechanical study	Baitner, Avi et al. (12)	Clinical Orthopaedics and Related Research	93
4	Biomechanical Evaluation of the Femoral Neck System in Unstable Pauwels III Femoral Neck Fractures: A Comparison with the Dynamic Hip Screw and Cannulated Screws	Stoffel, Karl et al. (16)	Journal of Orthopaedic Trauma	89
5	Biomechanical rationale for implant choices in femoral neck fracture fixation in the non-elderly	Panteli, Michalis et al. (6)	Injury-International Journal of the Care of the Injured	77
6	A Comparative Biomechanical Analysis of Fixation Devices for Unstable Femoral Neck Fractures: The Intertan Versus Cannulated Screws or a Dynamic Hip Screw	Rupprecht, Martin et al. (15)	Journal of Trauma-Injury Infection and Critical Care	77
7	Fracture Morphology of High Shear Angle “Vertical” Femoral Neck Fractures in Young Adult Patients	Collinge, Cory A. et al. (13)	Journal of Orthopaedic Trauma	64
8	An update on the Pauwels classification	Shen, Min et al. (3)	Journal of Orthopaedic Surgery and Research	41
9	Perception of Garden's classification for femoral neck fractures: an international survey of 298 orthopaedic trauma surgeons	Zlowodzki, M. et al. (4)	Archives of Orthopaedic and Trauma Surgery	39
10	Using a modified Pauwels method to predict the outcome of femoral neck fracture in relatively young patients	Shenghao Wang et al. 2015	Injury-International Journal of the Care of the Injured	34

### 3.6 Research directions and key words in this field

**Figure 4:**
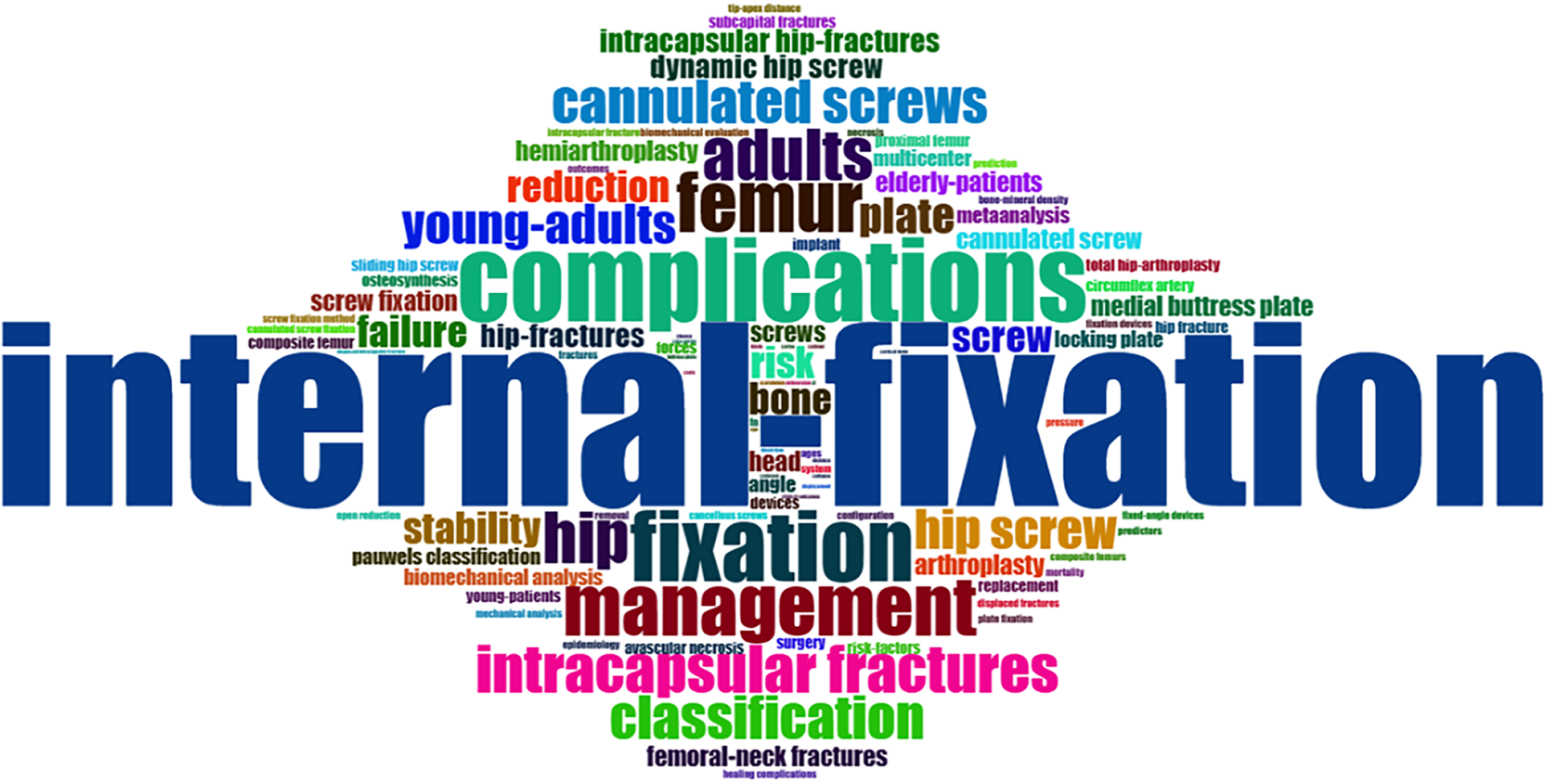
The high-frequency keywords in Pauwels III femoral neck fractures research field. The size of keywords was positively correlated with their frequency of occurrence in field of research. The higher the occurrence frequency was, the more prominent the keywords would be.

Figure 4 showed high-frequency keywords in the field regarding to Pauwels III femoral neck fractures. The size of keywords was positively correlated with their frequency of occurrence in field of research. The higher the occurrence frequency was, the more prominent the keywords would be. Clearly, “Internal-fixation” was the most eye-catching one, followed by “complications”, “managemment”, “intracapsular fracture”, “classification”, “young-adults”, “cannulated screws” and so on.

**Figure 5:**
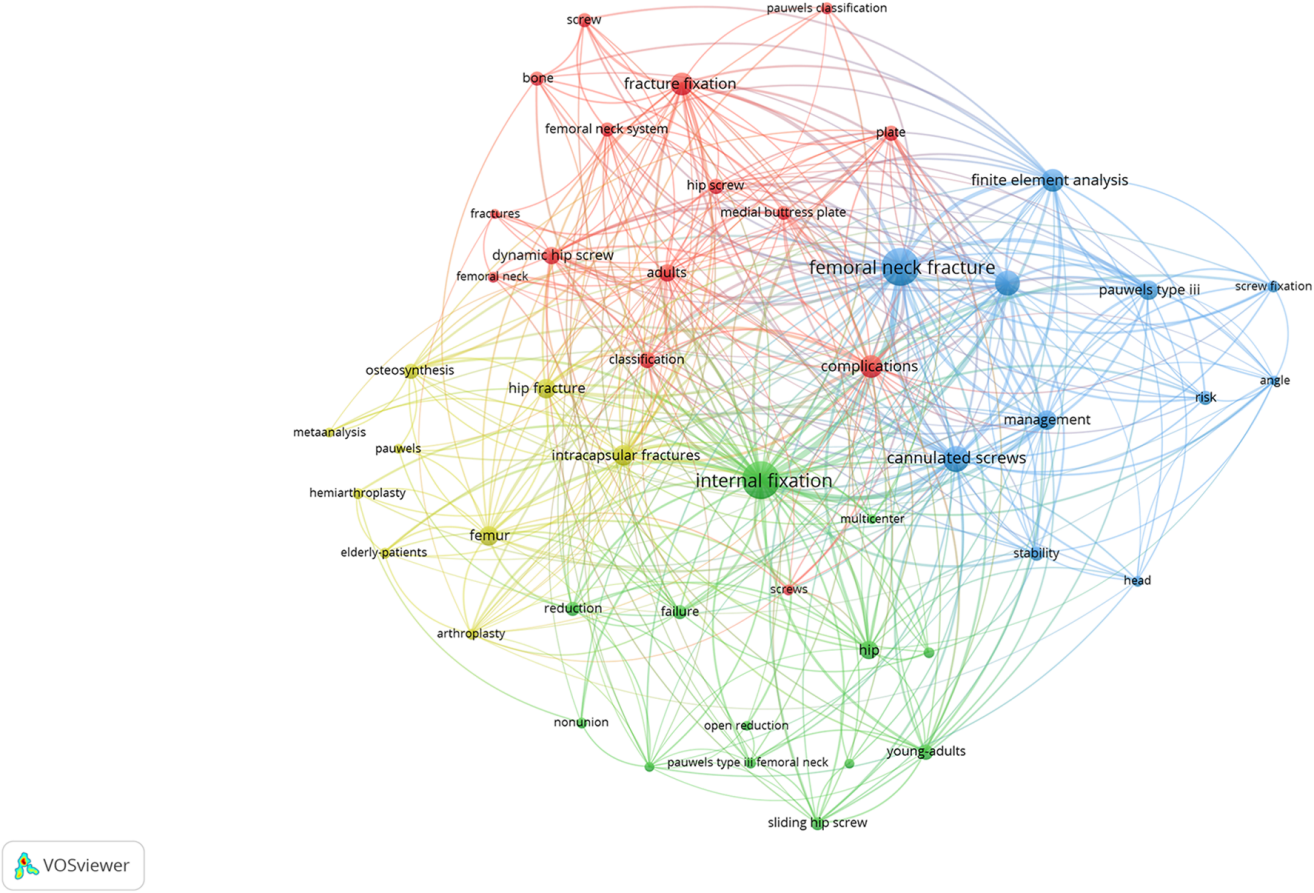
Keywords co-occurrence analysis in the network clustering view using VOSviewer. This study simplified and merged the keywords with similar senses to a certain extent.

By continuously adjusting parameters in VOSviewer, an ideal visual map was obtained, which intuitively displayed whether there was a co-occurrence relationship between keywords. The larger the node was, the more literature could be involved in that node's keywords. In the network clustering view, keywords were clustered according to co-occurrence relationships using different colors. The fact that keywords of the same color co-occurred more remarkable indicates that these research fields were related. In Figure 5, the 4 clusters marked by 4 colors symbolized 4 comparatively independent research dimensions. In Table 7, the details of top 5 keywords with high occurrence and total link strength in each cluster were listed.

**Table 7:** The top 5 keywords with high occurrence and total link strength (TLS) in each cluster marked by 4 different colors in figure 5.

Cluster	Main keywords	Occurrence (*n*)	TLS
Cluster 1 (blue)			
	Femoral neck fracture	67	352
	Cannulated screws	28	182
	Pauwels type III	20	132
	Finite element analysis	21	108
	Management	15	98
Cluster 2 (red)			
	Plate	23	139
	Complication	22	131
	Hip screw	23	128
	Classification	17	86
	Femoral neck system	9	51
Cluster 3 (green)			
	Internnal fixation	61	349
	reduction	12	78
	young adults	10	67
	Failure	8	49
	nonunion	5	27
Cluster 4 (yellow)			
	Intracapsular fracture	18	113
	Hip	14	70
	arthroplasty	10	59
	Osteosynthesis	10	59
	Elderly-patients	5	33

### 3.7 Global topic trends of the research area

**Figure 6:**
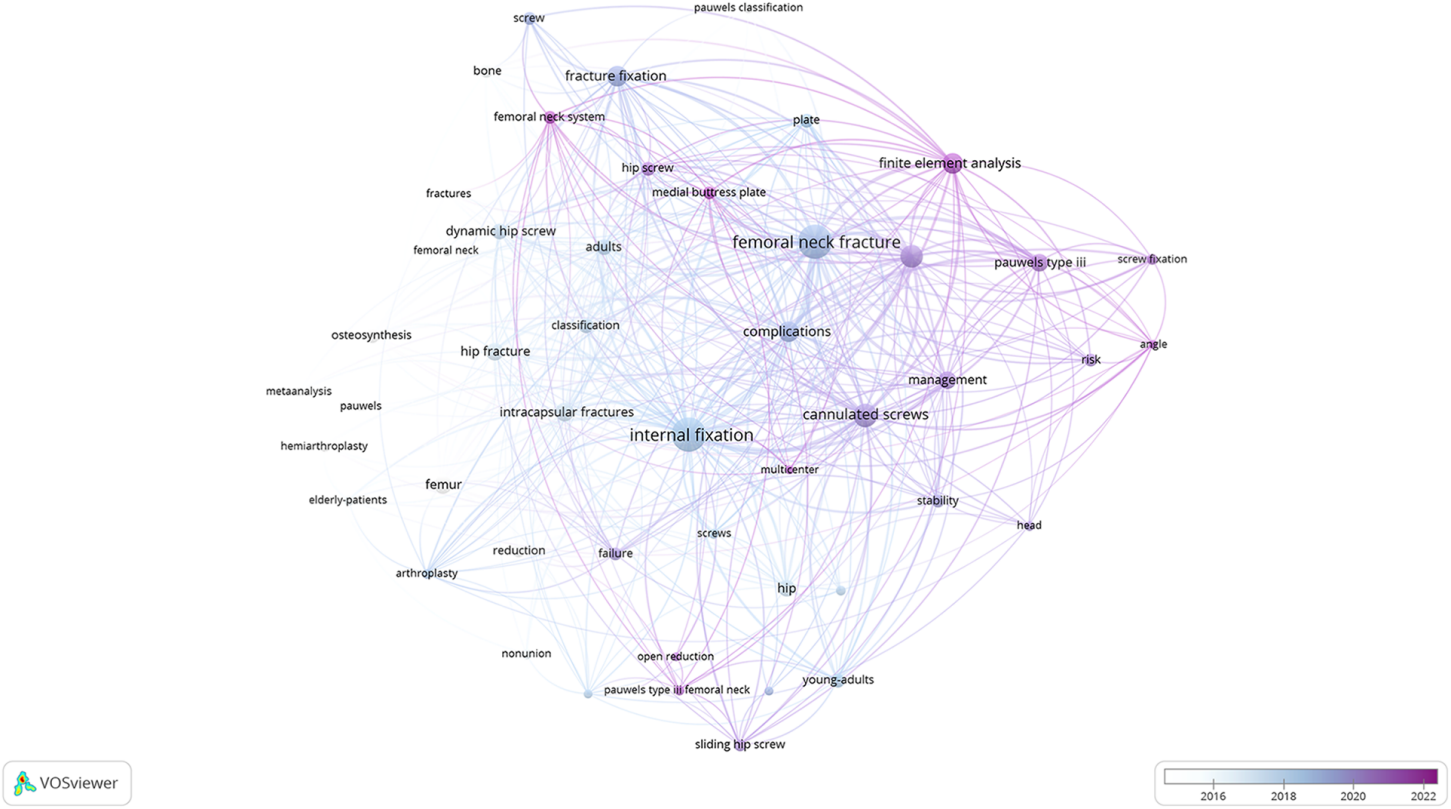
The overlay label view of VOSviewer co-occurrence analysis. The depth of keywords colors represented the average time in which they appeared. The darker the color, the newer the research direction.

In the overlay label view of VOSviewer co-occurrence analysis, the depth of keywords colors represented the average time in which they appeared. The darker the color, the newer the research direction. Thus, this timeview visual map (Figure 6) could reflect the current hot research fields, in which we could see clearly “finite element analysis”, “femoral neck system”, “hip screw”, “medial buttress plate” and “open reduction” were representatives.

**Figure 7:**
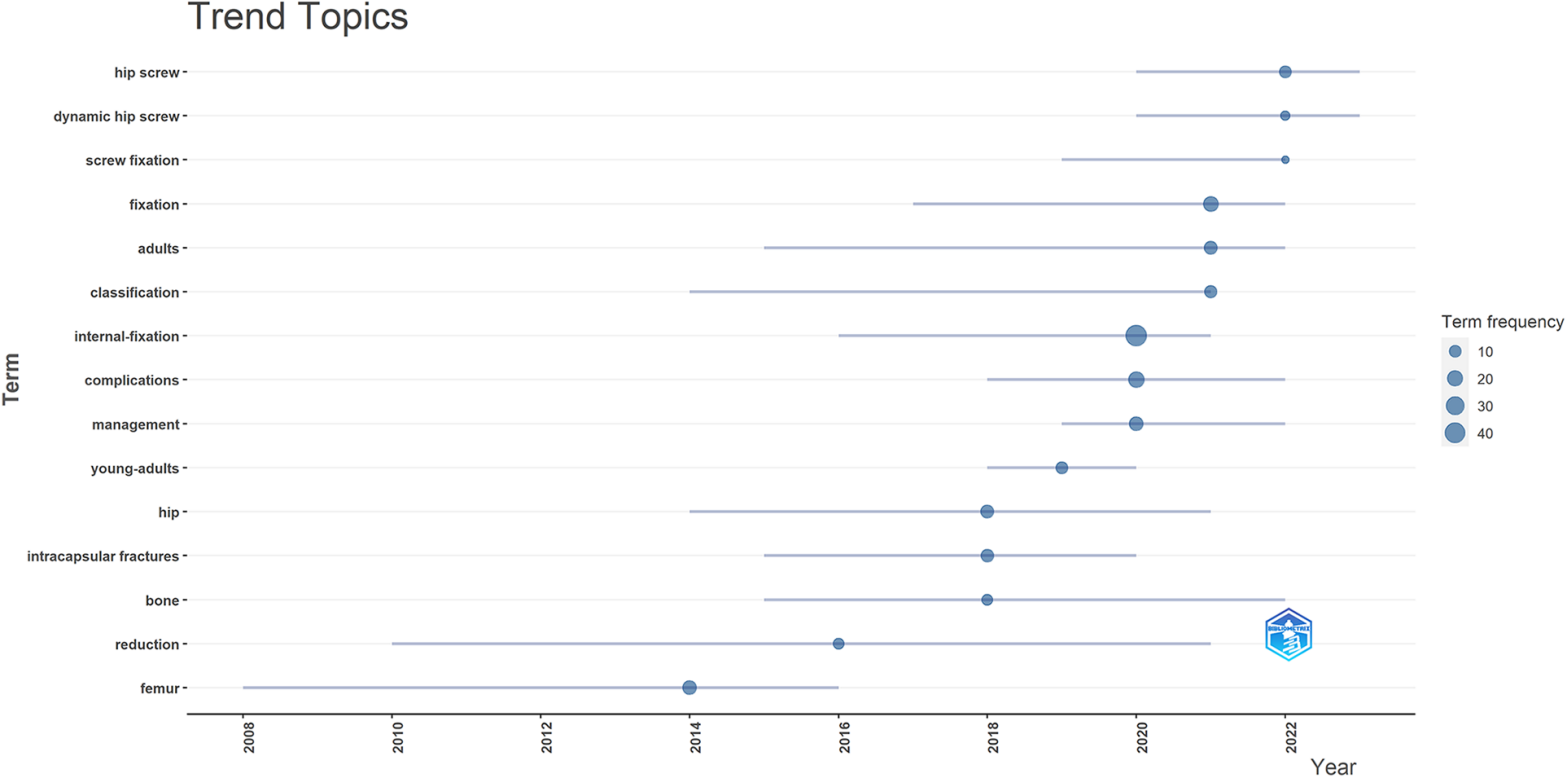
The time span and frequency of each topic about Pauwels III femoral neck fractures research. This figure showed the top 15 terms with relatively high frequency of occurrence, covering a total time span of 2008 to 2023.

Trend topics, processed by R package, could explore which directions in this field were worth studying. In Figure 7, the abscissa represented the year, which each term had a line segment parallel to. The length of the line segment was on behalf of the time span of the term occurrence, and the position of the dot on the line segment stood for the time point at which the term appeared most frequently. The larger the dot, the higher the frequency. The ordinate sorted these terms according to the time point of each dot. Therefore, the closer the term was to the upper right, the hotter the research direction would be. As could be seen from the figure, current popular research directions in this field were “hip screw”, “dynamic hip screw” and “screw fixation”. Meanwhile, within the time range of our study, “internal-fixation” had the highest frequency of research among all the terms, and “reduction” had the longest research span of more than 10 years.

**Figure 8:**
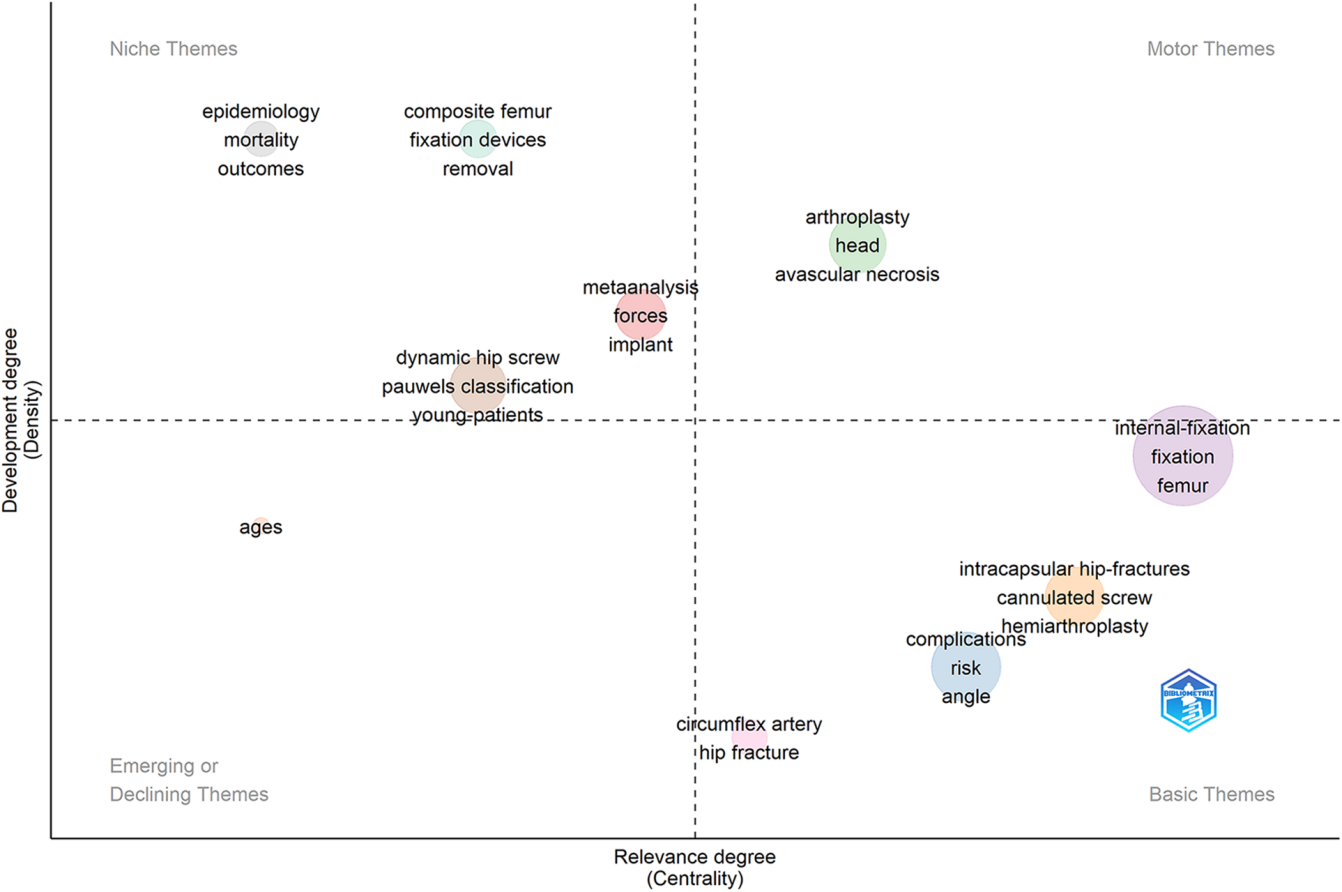
The thematic map of keywords concerning Pauwels III femoral neck fractures.

In order to further grasp the research status of each theme, we utilized R package to process these themes again. In Figure 8, the horizontal axis of the thematic map represented the centrality (relevance degree), and the vertical axis indicated the density (development degree). The higher the centrality, the more significant the theme was to our research field, and the higher the density, the more attention of the theme had acquired in the field. The circles of different colors each involved several highly correlated keywords. The size of each circle was positively correlated with the frequency of its relevant theme. Different themes were located in the map, divided into different quadrants by these 2 coordinate axes. The first quadrant (motor themes quadrant) was the worthiest research area currently, relying on these themes’ significance as well as well-development, such as “arthroplasty” and “avascular necrosis”. The second quadrant (niche themes quadrant) indicated that although the development of those themes was fine, for instance, “epidemiology”, “composite femur” and “fixation devices", they did not seem to have much impact on our research field. The third quadrant (emerging or declining themes quadrant) was on behalf of a comparatively marginal field, as a result of those themes’ novel or transient research status, like “ages”. The fourth quadrant (basic themes quadrant) was the representative of small but refined research directions, which carried weight in research area with not yet promising development. To some extent, these themes could predict the research trend of this field, for example, “internal-fixation”, “cannulated screw”, and “complications”.

## 4 Discussion

Compared to the publication volume in the first year, 1999, the publication volume in 2022 had exceeded its limit by more than 10 times. Pauwels type III femoral neck fractures were receiving increasing attention and input from scientists around the world, which indirectly validated the value and necessity to clarify the global research status and predict the research trends.

### 4.1 Current research distribution and quality analysis

By describing the country distribution, it could be visually seen that China ranked first in terms of publication production. The countries with high publication production were concentrated in developing countries with strong economic strength and developed countries, while economically underdeveloped regions such as Southeast Asia and Africa showed significant gaps, indicating that the economic strength of countries or regions played an important role in promoting the development of scientific research. Moreover, the top five research institutions with the largest production in this field belonged to China, with over half of the top ten research institutions in the world in terms of total publication volume belonging to China. Therefore, China has made the greatest scientific research contribution among countries in the research field of Pauwels III femoral neck fractures.

Related to the high production of publications, China maintained a leading position globally with regard to total citation and H-Index. However, the average citation of publications in the field of Pauwels III femoral neck fractures in China was significantly lower than that of developed countries, especially Switzerland. Based on a minuscule population, Switzerland's publications had an average citation of 42.7, ranking first in the world and far higher than China. This was closely related to its possession of the AO Foundation, the world's most prestigious academic organization in the realm of orthopaedics. As was known to all, the AO principles for fracture therapy published by the AO Foundation were known as the Bible of orthopedic doctors. Meanwhile, Switzerland's developed economic base was also a fatal reason for conducting high-quality academic research. About high-quality academic research, this study indicated it was convinced that there was a high probability that the excellent literature published in the future in this field would come from *Injury International Journal of the Care of the Injured* and *Journal of Orthopaedic Trauma*, which deserved our continuous attention and learning to obtain more cutting-edge academic trends.

Through keywords co-occurrence analysis, this study divided the research into four clusters. Among them, the keywords in the blue cluster chiefly included traditional therapy strategies represented by cannulated screws internal fixation and finite element analysis of the treatment methods for Pauwels III femoral neck fractures. The red cluster mainly displayed Pauwels classification of femoral neck fractures and other internal fixation methods besides cannulated screws for Pauwels III femoral neck fractures. The green cluster primarily introduced the conventional treatment principles and the outcome prediction for femoral neck fractures in young people, including keywords such as “reduction”, “internal fixation”, “nonunion”, “young people”, etc. While the content of the yellow cluster mostly focused on the therapy decisions in elderly patients, containing the keywords such as “intracapsular fractures”, “arthroplasty”, “elderly people”, etc. Moreover, both “reduction” and “internal fixation” had obtained extremely prominent attention in this field based on trend topics. As was shown in the keyword graph, “internal fixation” had proved to be the most popular keyword in current research. Thus, it can be seen that reduction and internal fixation provided quite a fundamental and precise treatment principle for Pauwels III femoral neck fractures for young patients.

### 4.2 Analysis of research trends in Pauwels III femoral neck fractures

By analyzing the heat of keywords and trend topics, this study indicated the current research hotspots for Pauwels III femoral neck fractures are the improvement and innovation of internal fixation methods for Pauwels III femoral neck fractures together with the corresponding finite element analysis, as well as the research on postoperative complications.

Currently, closed reduction is still the mainstream method for young people with Pauwels type III femoral neck fractures, while open reduction is gradually receiving academic attention. A two-year study on 150 patients with femoral neck fractures, all of whom went through closed reduction and percutaneous cannulated threaded screws therapy indicated that the incidence of avascular necrosis was 18%, which reflected that reduction quality is one of the key factors determining prognosis (18). Besides, Jason Halvorson et al. (19) believed that, except for non displacement fractures and severely comminuted fractures that cannot be anatomically reduced, open reduction should be the preferred choice for most young patients to realize anatomical reduction. However, a meta-analysis found that there were no statistical differences in the incidence of non union, avascular necrosis, and overall complications between open reduction and closed reduction for young patients with displaced femoral neck fractures, but acknowledged the lack of high-quality evidence (20). Accordingly, high-quality prospective study is still needed to further clarify the reduction methods for Pauwels III femoral neck fractures.

Cannulated screws internal fixation for femoral neck fractures has a few advantages including insertion over provisional pin fixation, low cost, low amount of bone removal, and can be inserted percutaneously in certain instances (21). However, its drawbacks include the incapability of providing stable angle support and could be prone to inversion and collapse, as well as the deficiency of control over compression between fractured ends, causing a trend of femoral neck shortening. In this circumstance, as two new technologies, the Smith & Nephew Conquest and Aesculap Targon systems, by force of the design that combines proximal femur locking plates and cannulated lag screws, can not only provide stable angular support for femoral neck fractures but also control the degree of continuous compression of the screws on the fractured ends. But currently, neither of the two improved technologies has been widely applied internationally (22). Besides that, in recent years, the exploration of reform in the configuration of cannulated threaded screws internal fixation has also been continuously in progress. By finite element analysis, Ru Yi Zhanga et al. (23) proposed the concept of oblique triangular configuration to acquire better stability to treat unstable femoral neck fractures. Jiantao Li et al. found that the triangular configuration formed by an upper partially threaded screw and two lower fully threaded screws could achieve better mechanical results between different configurations formed by fully or partially threaded screws (24), and when all using three parallel threaded screws for fixation, the inverted triangle configuration had a better performance (25). However, the effect of parallel cannulated screws in resisting shear displacement of the fractured fragments is not ideal, which explains the high rate of non union. A finite element analysis found that the cannulated screws with “F” configuration could preferably eliminate torsional and shear stresses while maintaining axial compressive stress at the fracture ends, especially combined with medial buttress plate (26). In summary, various improved protocols maintaining the application of cannulated screws have been explored to settle its postoperative complications, but clinical confirmation of their practicality is still indeed needed in the future.

Dynamic hip screw as an angle stabilization device not only can effectively prevent inversion and collapse in postoperation of femoral neck fractures, but promote fracture healing by compressing. Two meta-analysis found that dynamic hip screw was more suitable for treating vertical or displaced femoral neck fractures than cannulated threaded screws, because it had a lower risk of fracture non union and internal fixation failure (27, 28). However, dynamic hip screw usually only has one screw penetrating through the fracture, which will generate strong rotational force on femoral head when achieving intraoperative temporary satisfactory reduction, often leading to poor reduction condition and irreversible damage to the femoral head blood supply (29). A meta-analysis (30) found that dynamic hip screw had a higher rate of ischemic necrosis than cannulated threaded screws, which was statistically significant. In this regard, quite a few scholars suggested adding anti-rotation cannulated screws or blades to enhance the biomechanical stability of internal fixation when applying dynamic hip screw to treat femoral neck fractures (19, 31, 32). But a previous long-term cohort study showed that when using dynamic hip screws to treat vertical femoral neck fractures, even with the addition of anti-rotation screws, the reoperation rate was as high as 18% (33). Zhengqiang Li et al. (34) through a small-sample retrospective study found that the combination of dynamic hip screw and fibula bone graft could shorten healing time, reduce the rate of non union and avascular necrosis, compared to the combination of hip screw and an anti-rotation cannulated screw, providing a new therapy idea for Pauwels III femoral neck fracture in young people. Therefore, how to overcome the complications such as avascular necrosis when using dynamic hip screw has become the main problem currently faced.

FNS (femoral neck system) is a new type of internal fixation device that combines minimally invasive implantation and stable fixation. Biomechanical studies on unstable Pauwels type III femoral neck fractures had found that FNS had better biomechanical stability than three cannulated threaded screws (16, 35), and the system stability of FNS was equivalent to dynamic hip screw combined with anti-rotation screws (16). At present, FNS has gradually been applied in clinical practice, and corresponding retrospective studies have been completed, intended to compare the clinical effects of FNS with other implants, such as dynamic hip screw or three cannulated threaded screws. Recently, a retrospective study conducted by Konrad Schuetze et al. (36) found there was no significant difference in postoperative complications, the rate of internal fixture cutting out, and mortality between the two groups respectively treated with dynamic hip screw and FNS. A multicenter retrospective study indicated that in the treatment of Pauwels type III femoral neck fractures, FNS was superior to inverted triangular cannulated screws in terms of fracture healing time, Harris score, the degree of femoral neck shortening, as well as femoral neck axial angle changes, but there was no statistically significant difference in the incidence of femoral head necrosis and revision between the two (37). Therefore, FNS is expected to become a promising implant for the therapy of unstable femoral neck fractures, while further clinical research is needed to confirm this viewpoint.

The characteristics of vertical femoral neck fractures in young patients typically include a wide-based caudal head and neck segment, with the distal end mostly located at different positions near the medial femoral calcar, and accompanied by varying degrees of comminuted fractures which mostly located in the inferior and posterior quadrants (13, 38). Hassan Mir et al. (39) proposed applying buttress plate internal fixation to vertical fractures of the femoral neck in young people for its ability to stabilize fractures and resist the shear forces. A finite element analysis showed that MBP (medial buttress plate) could relieve stress focus on the fixation implant, providing an additional load path for the fractured ends. When combined MBP with cannulated screws, the risk of fixation failure could be significantly reduced, especially under dynamic loading (40). However, a retrospective study (41) showed that the combination of MBP and cannulated screws did not significantly increase Harris scores in non-elderly patients with Pauwels II and III femoral neck fractures at 6 months and beyond after surgery. Meanwhile, a multicenter retrospective study and corresponding biomechanical experiments (42) illustrated that compared with simply utilizing dynamic hip screw and anti-rotation screw to fix vertical femoral neck fractures, the additional application of MBP did not show statistical differences in the incidence of femoral neck shortening, femoral neck axial angle changes, avascular necrosis, as well as nonunion. Consequently, based on previous retrospective research results, the clinical value of medial buttress plate may require further prospective research to verify.

### 4.3 Advantages and limitations

This study made a reasonable prediction of the research trends of Pauwels III femoral neck fractures through bibliometric analysis, which exploring more improved or innovative treatment methods so as to address the postoperative complications mainly caused by cannulated screws therapy. However, there still existed some limitations in this study. Firstly, all publications covered by this bibliometric study were only from WoSCC database, which restricted the source of research. Secondly, in this study, we only extracted publications in English, resulting in language bias. Finally, due to the relatively low citation frequency of the latest publications, their impact in this study may be underestimated. Therefore, in future research, we will extract publications from more databases, such as Pubmed, besides, we will incorporate research on more languages besides English, and focus on the academic impact of the latest publications.

## 5 Conclusions

Through bibliometric research and visual analysis, this study elucidated the global academic status of Pauwels III femoral neck fractures, so as to predict and analyze the research trends in this field. China has made the greatest scientific research contribution among countries, but its academic quality should be improved further. *Injury International Journal of the Care of the Injured* and *Journal of Orthopaedic Trauma* can serve as milestone journals in this research field, which are worthy of continuous attention and learning in the future to obtain more cutting-edge academic trends. There is currently no optimal therapy for internal fixation of Pauwels III femoral neck fractures, while improved or innovative treatment methods designed to address the postoperative complications mainly caused by cannulated screws therapy will be a future research hotspot. But based on the fact that currently most of these emerging methods are still in the stage of *in vitro* experiments or small-scale retrospective studies, it is expected that corresponding higher quality clinical studies may be gradually carried out to further verify their actual clinical value in the future.

## Data Availability

The original contributions presented in the study are included in the article/Supplementary Material, further inquiries can be directed to the corresponding author.
